# Transcriptional analysis of rat piriform cortex following exposure to the organophosphonate anticholinesterase sarin and induction of seizures

**DOI:** 10.1186/1742-2094-8-83

**Published:** 2011-07-21

**Authors:** Kimberly D Spradling, Lucille A Lumley, Christopher L Robison, James L Meyerhoff, James F Dillman

**Affiliations:** 1 Cell and Molecular Biology Branch, US Army Medical Research Institute of Chemical Defense (USAMRICD), 3100 Ricketts Point Road, Aberdeen Proving Ground, MD 21010-5400, USA; 2 Neurobehavioral Toxicology Branch, US Army Medical Research Institute of Chemical Defense (USAMRICD), 3100 Ricketts Point Road, Aberdeen Proving Ground, MD 21010-5400, USA; 3 US Army Center for Environmental Health Research, 568 Doughten Drive, Fort Detrick, MD 21702-5010, USA

**Keywords:** Nerve Agent, Chemical Warfare, Organophosphate, Sarin, Piriform Cortex, Seizure, Neuroinflammation, Cytokine, Microarray, Transcriptomics

## Abstract

**Background:**

Organophosphorus nerve agents irreversibly inhibit acetylcholinesterase, causing a toxic buildup of acetylcholine at muscarinic and nicotinic receptors. Current medical countermeasures to nerve agent intoxication increase survival if administered within a short period of time following exposure but may not fully prevent neurological damage. Therefore, there is a need to discover drug treatments that are effective when administered after the onset of seizures and secondary responses that lead to brain injury.

**Methods:**

To determine potential therapeutic targets for such treatments, we analyzed gene expression changes in the rat piriform cortex following sarin (O-isopropyl methylphosphonofluoridate)-induced seizure. Male Sprague-Dawley rats were challenged with 1 × LD_50 _sarin and subsequently treated with atropine sulfate, 2-pyridine aldoxime methylchloride (2-PAM), and the anticonvulsant diazepam. Control animals received an equivalent volume of vehicle and drug treatments. The piriform cortex, a brain region particularly sensitive to neural damage from sarin-induced seizures, was extracted at 0.25, 1, 3, 6, and 24 h after seizure onset, and total RNA was processed for microarray analysis. Principal component analysis identified sarin-induced seizure occurrence and time point following seizure onset as major sources of variability within the dataset. Based on these variables, the dataset was filtered and analysis of variance was used to determine genes significantly changed in seizing animals at each time point. The calculated p-value and geometric fold change for each probeset identifier were subsequently used for gene ontology analysis to identify canonical pathways, biological functions, and networks of genes significantly affected by sarin-induced seizure over the 24-h time course.

**Results:**

A multitude of biological functions and pathways were identified as being significantly altered following sarin-induced seizure. Inflammatory response and signaling pathways associated with inflammation were among the most significantly altered across the five time points examined.

**Conclusions:**

This analysis of gene expression changes in the rat brain following sarin-induced seizure and the molecular pathways involved in sarin-induced neurodegeneration will facilitate the identification of potential therapeutic targets for the development of effective neuroprotectants to treat nerve agent exposure.

## Background

Sarin (O-isopropyl methylphosphonofluoridate) is a toxic organophosphorus (OP) nerve agent that was first discovered on October 10, 1938, by German scientists who were originally tasked with synthesizing more potent pesticides [1]. The production and stockpiling of sarin and other chemical warfare agents (CWAs) was banned by the Chemical Weapons Convention of 1993. However, OP nerve agents still remain a threat in armed conflicts and terrorist attacks, such as the terrorist sarin gas attack on the Tokyo subway in 1995 by members of the Japanese Uhm-Shinrikiu cult; the attack resulted in injuries to more than 5,500 civilians and 12 deaths [2,3]. CWAs are likely to be a weapon of choice for many other terrorist organizations because they are relatively accessible or simple to produce, easy to transport, and can be delivered in mass quantities [4,5].

Like other OP nerve agents, sarin irreversibly inhibits acetylcholinesterase (AChE), causing an accumulation of acetylcholine (ACh) at cholinergic synapses. This ACh buildup results in a cholinergic crisis due to overstimulation of muscarinic and nicotinic receptors in the central and peripheral nervous system, including the neuromuscular junction [4,6,7]. A victim exposed to these CWAs initially experiences symptoms such as myosis, tightening of the chest, difficulty breathing, and a general loss of bodily functions. As symptoms progress, the victim suffers from convulsive spasms and seizures, which can lead to death if left untreated [4,6-10].

Current medical countermeasures to nerve agent intoxication include an anti-muscarinic (e.g., atropine) that blocks excess ACh at muscarinic receptors to alleviate parasympathetic overstimulation, an oxime (e.g., 2-pyridine aldoxime methylchloride, 2-PAM) to reactivate inhibited AChE molecules, and an anticonvulsant such as diazepam [6-8,11]. These therapeutics increase survival if administered within a short period of time following exposure, but they may not fully prevent neurological damage [2,6,10,12-14]. Previous studies have shown that the development of long-lasting seizure activity following nerve agent exposure is highly correlated with the occurrence of brain damage [6,15]. Survivors of nerve agent poisoning can experience long-term neurological and behavioral outcomes months or years following exposure [2]. Previous findings of Scremin et al. [16] revealed that sarin-exposed rats showed behavioral abnormalities up to 16 weeks post-exposure. To date, most of our understanding on this issue comes from studies performed on survivors of the Tokyo subway attack, and most of these findings encompass only the psychiatric sequelae due to the high prevalence of post-traumatic stress disorder [3]. More recently, Loh and colleagues [5] reported on the long-term cognitive sequelae of a soldier exposed to sarin gas by means of an improvised explosive device (IED) while he was deployed to Iraq in 2004. Testing performed ten months following exposure revealed that the victim suffered from reduced information processing speed, poor focused attention, and difficulty in motor coordination. Despite these studies, the long-term neurologic sequelae of nerve agent exposure are still unclear. Therefore, the molecular effects and biological pathways involved in nerve agent-induced neurodegeneration need to be examined to determine drug treatments that would be effective when administered after the onset of seizures and secondary responses that lead to brain injury.

Gene expression profiling is an effective approach for elucidating chemical toxicant mechanisms of action [17,18]. Oligonucleotide microarrays are commonly used to simultaneously measure the mRNA levels of thousands of genes in a cell and are capable of detecting even subtle changes in gene expression. Gene expression profiling has been successfully used to investigate the mechanisms of toxicity and resulting effects of various CWAs [19-24].

We performed gene expression profiling of the piriform cortex, one of the regions in the central nervous system to show massive early-onset tissue pathology from nerve agent-induced seizures [10,13,25], following sarin exposure in a rat model to identify the molecular effects involved in nerve agent-induced neurodegeneration. In the present manuscript, we have focused on the transcriptional responses observed in sarin-exposed seizing animals. The gene expression alterations seen in sarin-exposed non-seizing animals will be the focus of a future manuscript. Consistent with previous studies, gene ontology analysis revealed a strong inflammatory response following nerve agent-induced seizure onset [22-24,26-31]. Therefore, we have identified pro-inflammatory cytokines as potential molecular targets for the development of effective neuroprotectants following nerve agent exposure.

## Methods

### Sarin exposure

Male Sprague-Dawley rats (350-500 g) were obtained from Charles River Laboratories (Wilmington, MA). They were housed in a temperature-controlled room with a 12-h light/12-h dark cycle and given food and water *ad libitum*. The research for this study was conducted at the United States Army Medical Research Institute of Chemical Defense (USAMRICD; Aberdeen Proving Ground, MD), which is fully accredited by the Association for Assessment and Accreditation of Laboratory Animal Care, International. All of the animal procedures were approved by the Institute Animal Care and Use Committee at USAMRICD and conducted in accordance with the principles stated in the *Guide for the Care and Use of Laboratory Animals *(National Research Council, 1996) and the Animal Welfare Act of 1966 (P.L. 89-544), as amended.

PhysioTel^® ^F40-EET transmitters (Data Sciences International, St. Paul, MN) were surgically implanted into the animals to record bi-hemispheric cortical electroencephalogram (EEG) activity, body temperature, and gross motor activity throughout the study. After a two-week recovery period, the animals were challenged with 1 × LD_50 _sarin (108 μg/kg, sc) that was obtained and diluted in sterile saline at USAMRICD. One minute after seizure onset, animals were treated with atropine sulfate (2 mg/kg; Sigma-Aldrich, St. Louis, MO) and 2-PAM (25 mg/kg; Sigma-Aldrich), both administered in a single injection (im). Thirty minutes later, animals were given the anticonvulsant diazepam (10 mg/kg, sc; TW Medical Veterinary Supply, Austin, TX). Control animals received an equivalent volume of vehicle (saline), atropine sulfate, 2-PAM, and diazepam at time points corresponding to the injections administered to sarin-exposed animals. Approximately 50% of the animals challenged with sarin did not produce seizure activity following exposure. Drug treatments were not given to these sarin-exposed non-seizure animals or their matched controls. Naïve animals received no injections.

Behavioral observations were documented for each animal following exposure and placed in one of three categories (mild, moderate, or severe). The total was then calculated and graphed using the total number of toxic signs listed in the moderate (e.g., loss of posture, excessive salivation and/or lacrimation, and body tremors) and severe (e.g., complete loss of posture, clonic-tonic convulsions, and gasping) categories. These behavioral observations corresponded with the five stages of behavioral seizure intensity, which were rated using a modified Racine scale score [32]: stage 0 = baseline behaviors, including resting, grooming, chewing, and sleeping; stage 1 = inactivity, unusual posture, piloerection, frozen posture, clumsy motion, and excessive grooming or chewing; stage 2 = oral tonus, head bobs, and body tremors; stage 3 = forelimb myoclonus, prostrate body extension, and salivation or lacrimation; stage 4 = loss of posture, whole body tremors, rigidity, body jerks, and forelimb myoclonus followed by rearing; and stage 5 = complete loss of posture, falling or generalized tonic-clonic convulsions, and gasping. Statistical significance between sarin-exposed seizing animals and their controls was calculated using Student's *t*-test.

A total of 67 animals were euthanized by decapitation at 0.25, 1, 3, 6, or 24 h after seizure onset. The piriform cortex and trunk blood were immediately collected from each animal at the appropriate time point. Three animals were used for each experimental group (naïve, sarin-exposed non-seizure, non-seizure control [saline], sarin-exposed seizure, and seizure control [saline]) at each time point, with the exception of 1-h seizure control, 3-h sarin-exposed seizure, 24-h sarin-exposed non-seizure, and 24-h sarin-exposed seizure (n = 4). Each tissue was immediately snap-frozen in liquid nitrogen and stored at -80°C until use. The blood samples were centrifuged and erythrocyte AChE activity was determined according to a modified method by Ellman et al. [33] using acetylcholine as a substrate. Statistical significance between sarin-exposed seizing animals and their controls was calculated using Student's *t*-test with a sample size of *n *= 2 for 1-h sarin-exposed seizing, 3-h control and sarin-exposed seizing, and 6-h control animals. The sample size was *n *= 3 for 0.25-h control and sarin-exposed seizing, 1-h control, 6-h sarin-exposed seizing, and 24-h control animals. There was a sample size of *n *= 4 for 24-h sarin-exposed seizing animals. The determined enzymatic activity was expressed as units per milliliter (U/ml) blood.

### Sample preparation for microarray hybridization

Tissues were homogenized in RNeasy lysis buffer (QIAGEN, Valencia, CA) for three seconds at 5,000 rpm and ramped to 17,000 rpm for 30 sec using an Omni Programmable Digital Homogenizer (Omni International, Kennesaw, GA). Each homogenate was subsequently centrifuged for 10 min at 16,110 × g at room temperature, and the supernatant was transferred to a new microcentrifuge tube. Total RNA was then extracted and DNase I-treated using the RNeasy Mini Kit and RNase-Free DNase Set (QIAGEN) according to the manufacturer's protocol. The quantity and quality of the RNA was determined with a NanoDrop ND-1000 UV-vis spectrophotometer (Thermo Scientific, Wilmington, DE) and an Agilent Bioanalyzer (Agilent Technologies, Santa Clara, CA) throughout sample processing. Total RNA was processed for hybridization to GeneChip^® ^Rat Genome 230 2.0 oligonucleotide arrays (Affymetrix, Inc., Santa Clara, CA) using the BioArray Single-Round RNA Amplification and Biotin Labeling System (Enzo Life Sciences, Inc., Farmingdale, NY) as previously described [20]. In brief, 1 μg of total RNA was used to generate first strand cDNA by using a T7-linked oligo(dT) primer. After second strand synthesis, *in vitro *transcription was performed with biotinylated UTP and CTP for cRNA amplification. Biotinylated target cRNA generated from each of the 67 samples (3 naïve, 16 sarin-exposed non-seizure, 15 non-seizure control, 17 sarin-exposed seizure, and 16 seizure control) was processed according to the manufacturer's protocol using an Affymetrix GeneChip Instrument System http://affymetrix.com/support/technical/manual/expression_ manual.affx as previously described [20].

All microarray experiments were performed to comply with Minimal Information About a Microarray Experiment (MIAME) protocols and details can be found at the Gene Expression Omnibus (GEO) accessible through GEO Series accession number GSE28435. The data discussed in this publication have been deposited in the National Center for Biotechnology Information's Gene Expression Omnibus (GEO; http://www.ncbi.nlm.nih.gov/geo/) and are accessible through GEO Series accession number GSE28435.

### Microarray data analysis

Raw signal intensities from each GeneChip^® ^were imported into Partek Genomics Suite v6.4 (Partek, Inc., St. Louis, MO) and normalized using the robust multiarray averaging (RMA) algorithm [34]. Normalized data were analyzed by principal component analysis (PCA) [35] to identify patterns in the dataset and highlight similarities and differences among the 67 samples. The major sources of variability identified among the sarin-exposed seizing animals and their matched controls were used as grouping variables for analysis of variance (ANOVA). The calculated *p*-value and geometric fold change for each probeset identifier were imported into Ingenuity Pathways Analysis (IPA; Ingenuity^® ^Systems, http://www.ingenuity.com) to identify the biological functions, canonical pathways, and networks of genes significantly affected by sarin exposure. Biological functions are categories into which genes are classified based on their cellular or physiological role in a healthy or diseased organism. Genes may be classified into more than one biological function. A canonical pathway is a well-established signaling or metabolic pathway that is manually curated on the basis of published literature. Canonical pathways are fixed prior to data input and do not change upon data input. Networks are distinct from canonical pathways in that they are built *de novo *from input data based on known molecular interactions identified in the published scientific literature. To identify canonical pathways that were most significant to the dataset, molecules that met the designated *p*-value cutoff (≤ 0.05) and were associated with a canonical pathway in Ingenuity's Knowledge Base were considered for the analysis. The significance of the association between the dataset and the canonical pathway was measured in two ways: 1) A ratio of the number of molecules from the data set that mapped to the pathway divided by the total number of molecules that mapped to the canonical pathway was displayed. 2) Fisher's exact test was used to calculate a *p*-value determining the probability that the association between the genes in the dataset and the canonical pathway was explained by chance alone. To determine networks of genes significantly affected by sarin exposure, molecules were overlaid onto a global molecular network developed from information contained in Ingenuity's Knowledge Base. Networks of molecules were then algorithmically generated based on their connectivity. The functional analysis of a network identified the biological functions and/or diseases that were most significant to the molecules in the network. The network molecules associated with biological functions and/or diseases in Ingenuity's Knowledge Base were considered for the analysis. Right-tailed Fisher's exact test was used to calculate a *p*-value determining the probability that each biological function and/or disease assigned to that network is due to chance alone.

### Multiplexed RT-PCR

The GenomeLab Gene Expression Profiler (GeXP; Beckman Coulter, Inc., Brea, CA) genetic analysis system was used to measure the expression levels of 21 differentially expressed cytokines or chemokines (see Additional File 1) by multiplexed RT-PCR to validate the microarray data. Primers were designed using the eXpress Designer module of the GenomeLab eXpress Profiler software, with each primer consisting of 20 nucleotides of gene-specific sequence as well as a universal primer sequence. RT-PCR product sizes ranged from 151 to 351 nt with a 7-nt minimum separation size between each fragment (see Additional File 1). The custom multiplexed panel also contained glyceraldehyde 3-phosphate dehydrogenase (GAPDH) for normalization and an internal control gene (kanamycin resistance, Kan^r^).

RNA samples used in the microarray experiment and the GenomeLab GeXP Start Kit (Beckman Coulter, Inc.) were used for the RT-PCR reactions according to the manufacturer's protocol. The custom multiplex was first optimized by reverse primer dilution to attenuate the gene signals that were close to or above the linear detection limit of the GeXP system detector (130,000 RFU in raw data or 120,000 RFU in analyzed data) and to balance the signal of each peak within the multiplex reaction. The final concentrations of the reverse primers within the multiplex are shown in Additional File 2. Fifty nanograms of total RNA was reverse transcribed with the optimized reverse primer multiplex. Subsequently, 9.3 μl of cDNA from each RT reaction was transferred to the PCR reaction mix containing 20 nM of the forward primer set multiplex. All experiments included "no template" (i.e. without RNA) and "no enzyme" (i.e. without reverse transcriptase) negative controls to confirm the absence of peaks at the expected target sizes.

The fluorescently-labeled PCR products were diluted 1:20 in 10 mM Tris-HCl (pH 8), and 1 μl of each dilution was added to 38.5 μl sample loading solution along with 0.5 μl DNA size standard-400 (GenomeLab GeXP Start Kit). The GeXP system was then used to separate the amplified PCR products based on size by capillary gel electrophoresis and to measure their fluorescent dye signal strength in arbitrary units (A.U.) of optical fluorescence, which is the fluorescent signal minus background. The multiplexed RT-PCR data were initially analyzed using the Fragment Analysis module of the GenomeLab GeXP system software, followed by the eXpress Analysis module of the eXpress Profiler software. First, the length or size of the products was determined using the Fragment Analysis module. The fragment data, peak height, and peak area information was then imported into the analysis module of the eXpress Profiler software where the fragments were compared to the expected PCR product sizes to identify each transcript.

The expression of each gene within a sample was normalized to GAPDH expression to minimize inter-capillary variation, and the normalized intensity of each replicate (n ≥ 3) was used to calculate an average intensity of each sample group (i.e. control or sarin-induced seizure at each time point). The fold expression difference between control and sarin-induced seizure samples was then evaluated for all genes at each time point and compared to the fold expression changes obtained by microarray analysis.

## Results

### Clinical manifestations of sarin exposure

**Figure 1:**
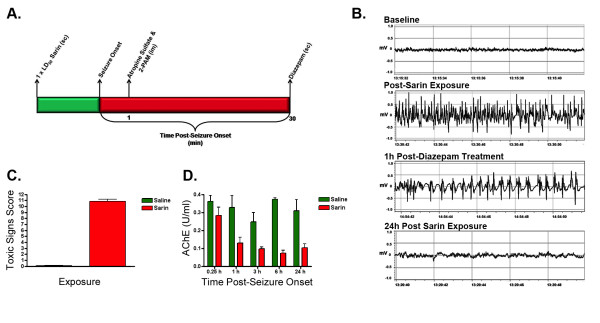
**Sarin exposure model and clinical manifestations**. (A) PhysioTel^® ^F40-EET transmitters were implanted into male Sprague-Dawley rats to monitor EEG activity, body temperature, and gross motor activity. After a two-week recovery period, animals were challenged with 1 × LD_50 _sarin, using saline as a vehicle. One minute post-seizure onset, animals were treated with 2 mg/kg atropine sulfate and 25 mg/kg 2-PAM (im). Thirty minutes later, animals were given 10 mg/kg diazepam (sc). Control animals received an equivalent volume of vehicle, atropine, 2-PAM, and diazepam. Drug treatments were not given to sarin-exposed non-seizure animals or their matched controls. The piriform cortex was extracted at 0.25, 1, 3, 6, and 24 h post-seizure onset, and at least three biological replicates were used per time point. (B) The EEG shows seizure activity following sarin exposure. It illustrates baseline EEG, seizure activity shortly after exposure, continued seizure activity 1 h after diazepam treatment, and subsiding seizure activity 24 h after sarin exposure. (C) The severity of toxic signs exhibited in seizing animals was significantly greater (*p *< 0.0001) than in controls. The graph indicates the total number of toxic signs listed in the moderate and severe categories. The control animals had an average toxic signs score of 0.08, and the seizing animals had an average score of 10.88. (D) The Ellman assay was used to measure the AChE activity for sarin-exposed seizing animals and their matched controls post-exposure. AChE activity was inhibited by 0.25 h post-seizure onset compared to control and continued to decrease over time until increasing slightly at 24 h. Statistically significant differences in AChE activity were seen at 6 h (*p *= 0.0007) and 24 h (*p *= 0.0168) post-seizure onset. The enzymatic activity at 0.25, 1, and 3 h was not significantly different, which is likely due to smaller sample sizes at 1 and 3 h.

Male Sprague-Dawley rats were challenged with 1 × LD_50 _sarin or saline (as control) as described under *Materials and Methods *(Figure 1A). EEG monitoring showed that seizures were induced in approximately 50% of the sarin-exposed animals, with a mean latency of 10.2 min (EEG example shown in Figure 1B). Behavioral seizure intensity was scored using a modified Racine scale [32]. The amount of moderate and severe toxic signs exhibited by sarin-exposed seizing animals was significantly greater (*p *< 0.0001) than their controls (Figure 1C), with the control animals having an average toxic signs score of 0.08 and the seizing animals having an average score of 10.88. Also, AChE activity was inhibited to a greater extent in sarin-exposed seizing animals than in their controls (Figure 1D). AChE activity was inhibited by 0.25 h after seizure onset compared to control and continued to decrease over time until increasing slightly at 24 h after seizure onset. Statistically significant differences in AChE activity were seen at 6 h (*p *= 0.0007) and 24 h (*p *= 0.0168) after seizure onset.

### Sarin exposure induces piriform cortex gene expression with the greatest effects in seizing animals

**Figure 2:**
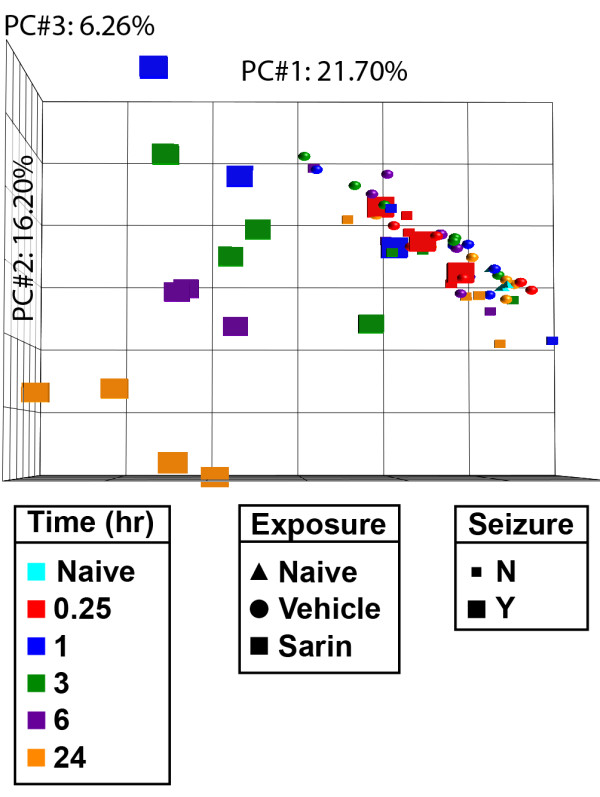
**Principal component analysis of piriform cortex samples**. PCA of gene expression profiles reveals partitioning of piriform cortex samples based on sarin-induced seizure occurrence and time point following seizure onset. Tissues were isolated at 0.25, 1, 3, 6, and 24 h after seizure onset and processed for oligonucleotide microarray analysis. The raw signal intensities were normalized using the RMA algorithm and visualized using PCA to identify major sources of variability in the data. Each point on the PCA represents the gene expression profile of an individual animal. Point shape corresponds to exposure condition, point color corresponds to the time after seizure onset at which the tissue was collected, and point size indicates absence or occurrence of sarin-induced seizure. The principal components in the three-dimensional plot represent the variability in gene expression levels seen within the dataset: PC#1 (*x*-axis) accounts for 21.70% of the variability in the data; PC#2 (*y*-axis) represents 16.20% of the variability; and PC#3 (*z*-axis) represents 6.26% of the variability in gene expression levels seen within the dataset.

Total RNA was isolated from the piriform cortex and processed for oligonucleotide microarray analysis to determine the molecular functions and biological pathways involved in sarin-induced toxicity. Raw data were normalized using the RMA algorithm [34] and analyzed by PCA (Figure 2) [35] to reduce the complexity of the multi-dimensional dataset. The resulting three-dimensional plot identified sarin-induced seizure occurrence and time after seizure onset (0.25, 1, 3, 6, or 24 h) as major sources of variability within the dataset. Each point on the PCA represents the gene expression profile of an individual animal, and the distance between any two points is a function of the similarity in gene expression profiles between those two samples. Therefore, samples that are near each other in the three-dimensional plot have similar gene expression profiles, whereas those that are farther apart have dissimilar gene expression responses to sarin-induced seizure over time. The naïve, saline-exposed controls, and sarin-exposed non-seizing animals partitioned together within the three-dimensional plot. However, the sarin-exposed seizing animals partitioned away from controls and clustered together based on time after seizure onset, with the 24-h seizing animals separated the furthest from controls. This indicates that the greatest differences in gene expression profiles exist between control and 24-h sarin-exposed seizing animals.

### Canonical pathways associated with inflammation identified as significantly altered in piriform cortex of sarin-exposed seizing animals

**Figure 3:**
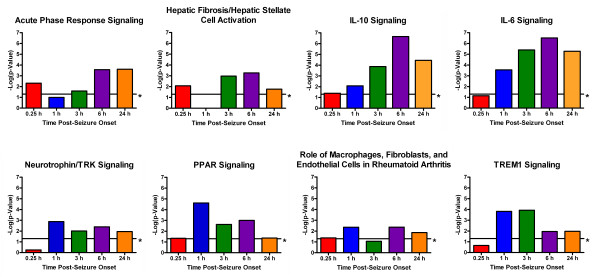
**Inflammatory-related pathways identified among the most significantly altered pathways in sarin-exposed seizing animals**. An ANOVA was performed to identify genes most significantly changed at each time point based on exposure (sarin vs. vehicle). The *p*-value and geometric fold change for each probeset were imported into IPA to identify the biological functions and canonical pathways most affected by sarin-induced seizure at each time point. Genes from the dataset that met the *p-*value cutoff and were associated with a canonical pathway in the IPA Knowledge Base were considered for the analysis. The significance of the association between the dataset and the canonical pathway was measured using a *p*-value that was calculated using Fisher's exact test to determine the probability that the association between the genes in the dataset and the canonical pathway was explained by chance alone. The calculated *p*-values for each time point are displayed as colored bars, with a threshold of 0.05 (or 1.3 when expressed as -log(p-value)) marked by an asterisk.

**Figure 4:**
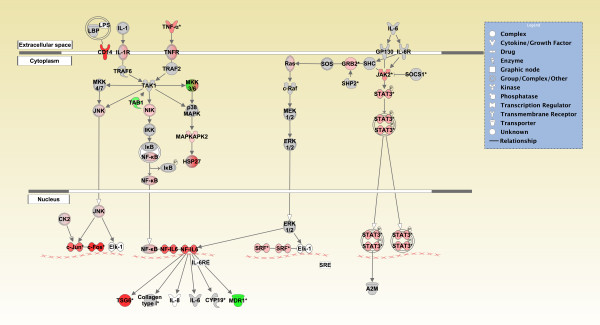
**Graphical representation of the IL-6 signaling pathway reveals significant gene expression changes following sarin-induced seizure**. Genes in the IL-6 signaling pathway at the 6 h time point for sarin-induced seizing animals are represented as nodes of various shapes to represent the functional class of the gene product, and the biological relationship between two nodes is represented as a line. The intensity of the node color indicates the degree of up- (red) or down- (green) regulation. Nodes shown in gray represent genes from the dataset that did not meet the *p*-value cutoff, and nodes shown in white represent genes that are in IPA's Knowledge Base but not in the dataset.

To determine the molecular effects of sarin-induced seizure occurrence, the data were filtered to examine gene changes at each time point following seizure onset (0.25, 1, 3, 6, or 24 h), and a one-way ANOVA was performed using exposure (saline vs. sarin) as a grouping variable. The calculated *p*-value and geometric fold change for each probeset ID from the arrays were imported into IPA to identify the biological functions and canonical pathways most affected by sarin-induced seizure at each time point. The top ≤ 800 genes that met the *p-*value cutoff (≤ 0.05) and were associated with a canonical pathway in the IPA Knowledge Base were considered for each analysis. Significant changes in gene expression were seen as early as 0.25 h after seizure onset (p ≤ 0.05, see Additional File 3) and progressively increased over the time course (1 h: p ≤ 4.35 × 10^-2^, 3 h: p ≤ 4.66 × 10^-3^, 6 h: p ≤ 2.54 × 10^-3^, 24 h: 6.25 × 10^-4^; see Additional Files 4, 5, 6 and 7). Gene ontology analysis revealed numerous biological functions and canonical pathways that were significantly altered by sarin-induced seizure. Canonical pathways associated with an inflammatory response (such as acute phase response signaling; hepatic fibrosis/hepatic stellate cell activation; interleukin (IL)-10 signaling; IL-6 signaling; neurotrophin/tyrosine kinase receptor [TRK] signaling; peroxisome proliferator-activated receptor [PPAR] signaling; role of macrophages, fibroblasts, and endothelial cells in rheumatoid arthritis; and triggering receptor expressed on myeloid cells 1 [TREM1] signaling) were among the most significantly altered across the five time points examined (Figure 3), and many of the same pro-inflammatory cytokines (tumor necrosis factor-α [TNF-α], IL-1β, IL-6) are represented in all of these pathways (as seen in the graphical representation of the IL-6 signaling pathway in Figure 4). Additionally, inflammatory response was among the top 25 significantly altered biological functions for each time point (see Additional Files 8, 9, 10,11 and 12).

### Biological functions and canonical pathways affected at 0.25 h after seizure onset

A multitude of biological functions and canonical pathways were significantly altered as early as 0.25 h following seizure onset. The list of biological functions varied, with cancer, cellular development, and organ development most significant. Cell death, cell-mediated immune response, humoral immune response, immune cell trafficking, and inflammatory response were also observed among the 25 most significantly affected functions (see Additional File 8). Unlike the later time points, a number of metabolic pathways were significantly modulated immediately after sarin-induced seizure. These included pathways involving amino acid metabolism (selenoamino acid metabolism and D-glutamine and D-glutamate metabolism), carbohydrate metabolism (inositol metabolism, which was the most significant pathway), energy metabolism (nitrogen metabolism), glycan biosynthesis and metabolism (chondroitin sulfate biosynthesis), and metabolism of complex lipids (phospholipid degradation). In addition to these metabolic pathways, a number of signaling pathways were modulated 0.25 h after seizure onset. The two most significantly altered signaling pathways were acute phase response signaling and hepatic fibrosis/hepatic stellate cell activation, both of which are involved in cytokine signaling and inflammation, followed by tight junction signaling, which plays a role in apoptosis signaling and cell cycle regulation. Other significantly modulated pathways involved in cytokine signaling include interferon signaling and IL-10 signaling (see Additional File 13).

### Biological functions and canonical pathways affected at 1 h after seizure onset

The number of significantly modulated genes and canonical pathways increased over time following sarin-induced seizure. The biological functions most affected 1 h after seizure onset were cell death, cellular growth and proliferation, gene expression, and cellular movement. As with the 0.25-h time point, inflammatory-related functions were among the top 25 significantly altered biological categories (immunological disease, inflammatory disease, immune cell trafficking, inflammatory response, and cell-mediated immune response) (see Additional File 9). Unlike the 0.25-h time point, no metabolic pathways were found to be significantly altered at 1 h after seizure onset, but an increasing number of signaling pathways were affected. The most significantly altered signaling pathway was PPAR signaling, which plays a role in nuclear receptor signaling and has been associated with inflammation (see Additional File 14). Among the other significant pathways were those involved in cytokine signaling (TREM1 signaling, IL-6 signaling, prolactin signaling, p38 mitogen activated protein kinase [MAPK] signaling, high-mobility group protein 1 [HMGB1] signaling, IL-10 signaling, IL-2 signaling, granulocyte-macrophage colony-stimulating factor [GM-CSF] signaling, nuclear factor kappa-light-chain-enhancer of activated B cells [NF-κB] signaling, IL-12 signaling and production in macrophages, airway pathology in chronic obstructive pulmonary disease, and IL-9 signaling) and nervous system signaling (neurotrophin/TRK signaling, cholecystokinin/gastrin-mediated signaling, circadian rhythm signaling, and gonadotropin-releasing hormone [GNRH] signaling) (see Additional File 14).

### Biological functions and canonical pathways affected at 3 h after seizure onset

At 3 h after seizure onset, an increase in the number and level of significance of genes and canonical pathways was observed. The most significant biological functions were cellular growth and proliferation, cell death, and embryonic development (see Additional File 10). As with the previous two time points, a number of inflammatory functions were listed in the top 25 categories (cell-mediated immune response, immunological disease, and inflammatory disease) (see Additional File 10). Like the 1 h time point, no metabolic pathways were found to be significant, but the number of significant signaling pathways increased. The most significant pathways 3 h after seizure onset were IL-6 signaling, TREM1 signaling, IL-10 signaling, and HMGB1 signaling, which are all involved in cellular immune responses and cytokine signaling. Among the other significant pathways were those involved in cellular stress and injury (ataxia telangiectasia mutated protein [ATM] signaling, p38 MAPK signaling, endoplasmic reticulum stress pathway, hypoxia signaling in the cardiovascular system, and nuclear factor erythroid 2-related factor 2 [NRF2]-mediated oxidative stress response) (see Additional File 15).

### Biological functions and canonical pathways affected at 6 h after seizure onset

At 6 h after seizure onset, the most significant molecular functions were cell death and inflammatory response. These were then followed by cellular growth and proliferation, tissue development, connective tissue development and function, and skeletal and muscular system development and function (see Additional File 11). Three metabolic pathways were significantly modulated at 6 h following sarin-induced seizure. These included pathways involved in amino acid metabolism (valine, leucine, and isoleucine degradation and β-alanine metabolism) and glycan biosynthesis and metabolism (keratan sulfate biosynthesis). The most significant signaling pathways at 6 h after seizure onset included many that are associated with an inflammatory response and are involved in cytokine signaling. The five most significant pathways were IL-6 signaling, IL-10 signaling, type I diabetes mellitus signaling (an autoimmune disease involving many of the same pro-inflammatory cytokines seen in the other top pathways), acute phase response signaling, and p38 MAPK signaling (see Additional File 16).

### Biological functions and canonical pathways affected at 24 h after seizure onset

At 24 h following seizure onset, which was the latest time point included in the study, inflammation was still among the 25 most significant functions identified (inflammatory response, inflammatory disease, and immune cell trafficking). Also included in the most significant biological functions were cellular growth and proliferation, cellular movement, and cellular development (see Additional File 12). Metabolic disease was among the top 25 functions, and taurine and hypotaurine metabolism was among the significant pathways. At 24 h, the most significant canonical pathways were similar to those seen at 6 h after seizure onset, with IL-6 and IL-10 signaling being the two most significant pathways (see Additional File 17). The third pathway identified was integrin-linked kinase (ILK) signaling, which is involved in cellular growth, proliferation, and development. In addition to IL-6 and IL-10 signaling, other significant pathways involved in cellular immunity and cytokine signaling included acute phase response signaling, macrophage migration inhibitory factor (MIF) regulation of innate immunity, toll-like receptor signaling, dendritic cell maturation, and TREM1 signaling (see Additional File 17).

### Top *de Novo *networks modulated in piriform cortex in sarin-exposed seizing animals

**Figure 5:**
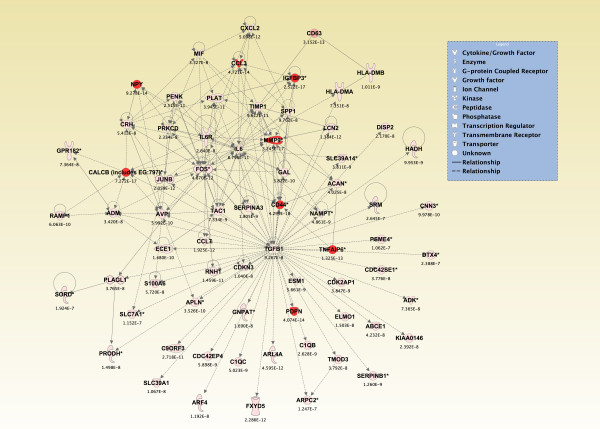
**Seizure-induced alteration of cell-to-cell signaling and interaction, inflammatory response, and cellular movement gene network**. A two-way ANOVA was performed to identify genes most significantly changed based on exposure (saline or sarin) and time after seizure onset interaction. Each probeset ID and corresponding false discovery rate (FDR) corrected p-value was imported into IPA and mapped to its corresponding gene in the IPA Knowledge Base. A p-value cutoff of 3.046 × 10^-7 ^was set to limit the number of molecules considered for the analysis and identify genes whose expression was significantly differentially regulated. Genes meeting the cutoff criteria were overlaid onto a global molecular network developed from information within the IPA Knowledge Base, and the networks were then algorithmically generated based on their connectivity. Graphical representation of the network reveals significant changes in gene expression due to sarin-induced seizure. Genes are represented as nodes of various shapes to represent the functional class of the gene product, and the biological relationship between two nodes is represented as a line. The intensity of the node color indicates the degree of differential expression.

**Figure 6:**
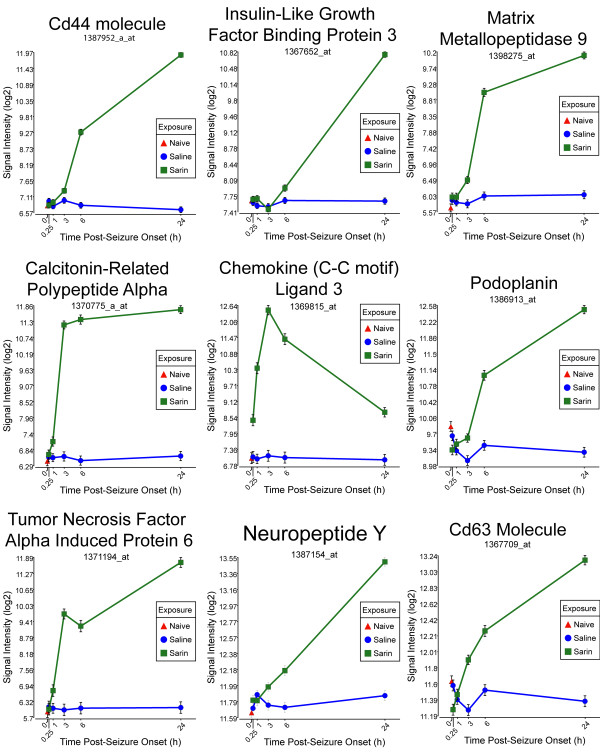
**Seizure-induced alteration of significant genes from cell-to-cell signaling and interaction, inflammatory response, and cellular movement gene network**. The top nine genes within the network shown in Figure 5 significantly altered by sarin-induced seizure. Expression of each gene was up-regulated after seizure onset and continued to rise over the 24-h time period. An exception to this trend was seen with chemokine (C-C motif) ligand 3 (CCL3) and tumor necrosis factor alpha induced protein 6 (TNFAIP6), where expression modestly dropped at 6 h. CCL3 expression continued to drop at 24 h, while TNFAIP6 expression went back up at 24 h after seizure onset.

**Figure 7:**
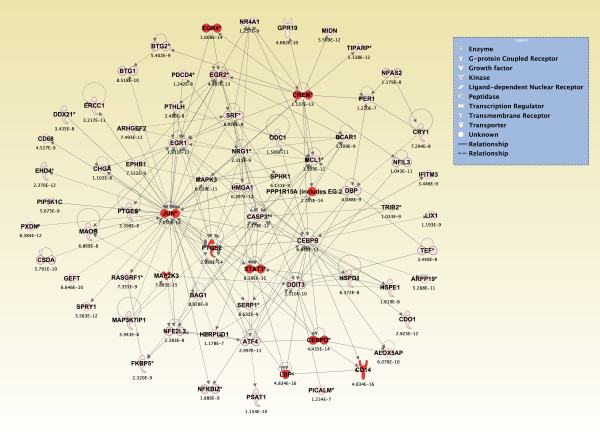
**Seizure-induced alteration of cell death, cellular development, and cellular function and maintenance gene network**. As detailed in Figure 5, a two-way ANOVA was performed to identify genes most significantly changed based on exposure and time after seizure onset. Genes meeting a p-value cutoff of 3.046 × 10^-7 ^were overlaid onto a global molecular network developed from information within the IPA Knowledge Base, and the networks were then algorithmically generated based on their connectivity. Graphical representation of the network reveals significant changes in gene expression due to sarin-induced seizure. Genes are represented as nodes of various shapes to represent the functional class of the gene product, and the biological relationship between two nodes is represented as a line. The intensity of the node color indicates the degree of differential expression.

**Figure 8:**
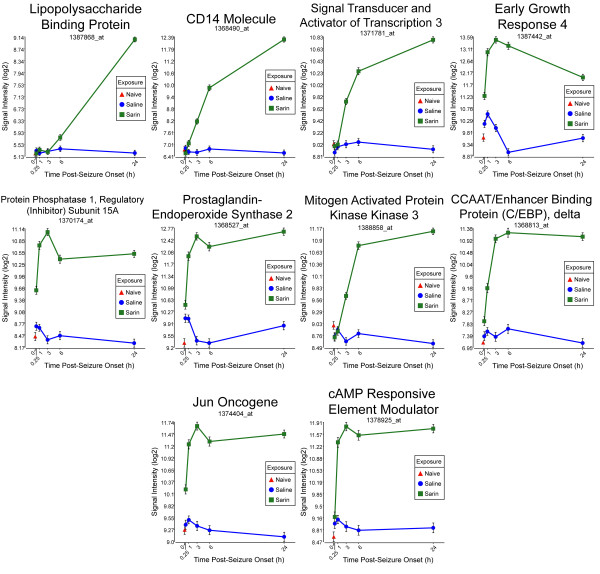
**Seizure-induced alteration of significant genes from cell death, cellular development, and cellular function and maintenance gene network**. The top ten genes within the network shown in Figure 7 significantly altered by sarin-induced seizure. Expression of CD14 molecule, signal transducer and activator of transcription 3 (STAT3), mitogen activated protein kinase kinase 3 (MAP2K3), and lipopolysaccharide binding protein (LBP) was up-regulated at 1, 3, 3, and 6 h, respectively, after seizure onset and continued to rise over the 24-h time period. Early growth response 4 (EGR4) expression was immediately up-regulated at 0.25 h after seizure onset, peaked at 3 h, and then decreased over the remainder of the time course. A similar expression pattern was seen for protein phosphatase 1 regulatory (inhibitor) subunit 15A (PPP1R15A), prostaglandin-endoperoxide synthase 2 (PTGS2), JUN oncogene (JUN), and cAMP responsive element modulator (CREM) where expression levels peaked at 3 h, dropped slightly at 6 h, and increased at 24 h after seizure onset. CCAAT/enhancer binding protein (C/EBP) δ increased from 0.25 h to 6 h after seizure onset and had a very slight drop at 24 h.

**Figure 9:**
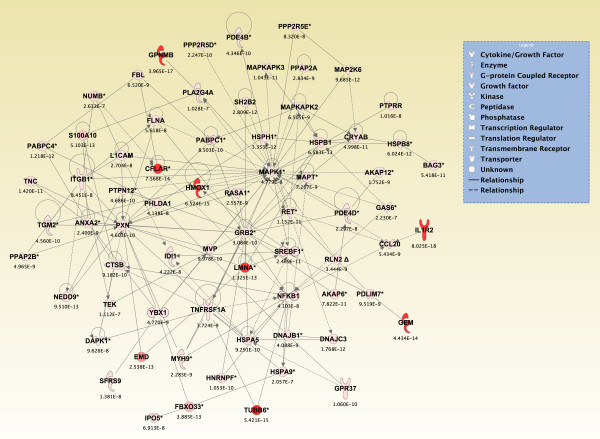
**Seizure-induced alteration of cellular movement, cell death, and cell morphology gene network**. As detailed in Figure 5, a two-way ANOVA was performed to identify genes most significantly changed based on exposure and time after seizure onset. Genes meeting a p-value cutoff of 3.046 × 10^-7 ^were overlaid onto a global molecular network developed from information within the IPA Knowledge Base, and the networks were then algorithmically generated based on their connectivity. Graphical representation of the network reveals significant changes in gene expression due to sarin-induced seizure. Genes are represented as nodes of various shapes to represent the functional class of the gene product, and the biological relationship between two nodes is represented as a line. The intensity of the node color indicates the degree of differential expression.

**Figure 10:**
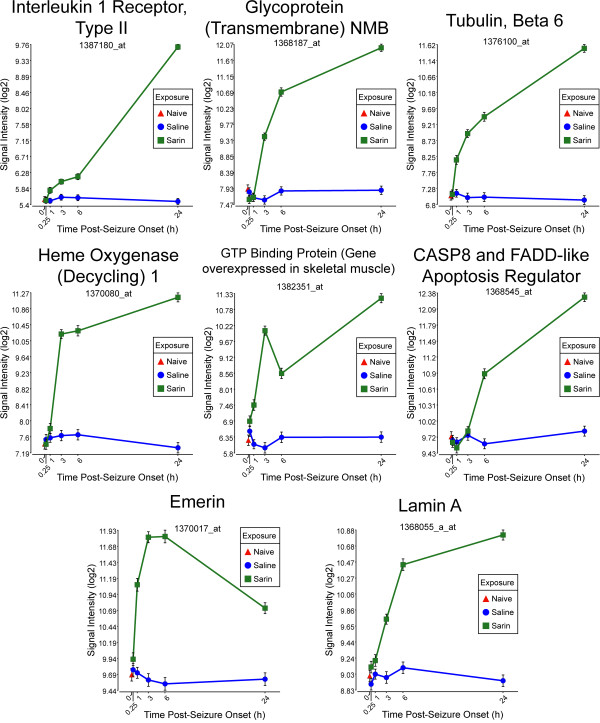
**Seizure-induced alteration of significant genes from cellular movement, cell death, and cell morphology gene network**. The top eight genes within the network shown in Figure 9 significantly altered by sarin-induced seizure. Interleukin 1 receptor type II (IL1R2), glycoprotein (transmembrane) NMB (GPNMB), tubulin β6 (TUBB6), heme oxygenase (decycling) 1 (HMOX1), and lamin A (LMNA) expression increased over the 24-h time course. GTP binding protein (gene overexpressed in skeletal muscle) (GEM) increased from 0.25 to 3 h after seizure onset, decreased at 6 h, and increased again at 24 h. The expression level of emerin (EMD) peaked at 3 and 6 h after seizure onset and dropped at 24 h. CASP8 and FADD-like apoptosis regulator (CFLAR) had a modest decrease in expression at 1 h after seizure onset and then increased in expression over the remainder of the time course.

**Figure 11:**
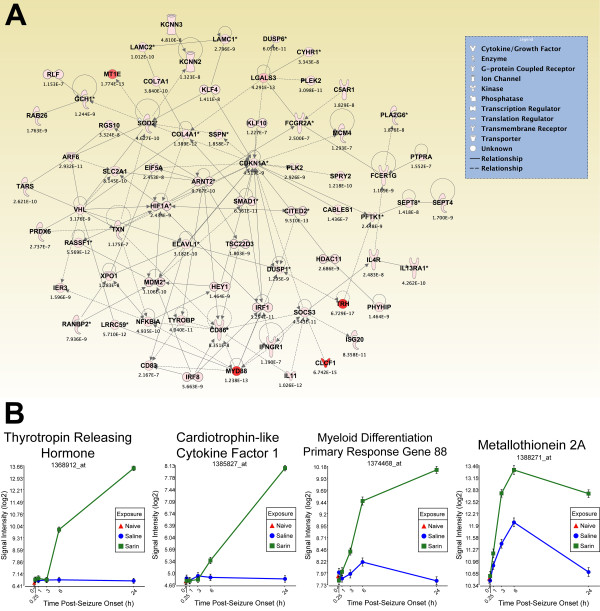
**Seizure-induced alteration of cellular growth and proliferation, cellular development, and lipid metabolism gene network**. As detailed in Figure 5, a two-way ANOVA was performed to identify genes most significantly changed based on exposure and time after seizure onset. Genes meeting a p-value cutoff of 3.046 × 10^-7 ^were overlaid onto a global molecular network developed from information within the IPA Knowledge Base, and the networks were then algorithmically generated based on their connectivity. (A) Graphical representation of the network reveals significant changes in gene expression due to sarin-induced seizure. Genes are represented as nodes of various shapes to represent the functional class of the gene product, and the biological relationship between two nodes is represented as a line. The intensity of the node color indicates the degree of differential expression. (B) Top four genes within network significantly altered by sarin-induced seizure. The expression levels of thyrotropin releasing hormone (TRH), cardiotrophin-like cytokine factor 1 (CLCF1), and myeloid differentiation primary response gene 88 (MYD88) increased during the time period examined with peak expression at 24 h after seizure onset. Metallothionein 2A (labeled MT1E in IPA network) expression peaked at 6 h after seizure onset and dropped at 24 h in both the control and sarin-exposed animals.

**Figure 12:**
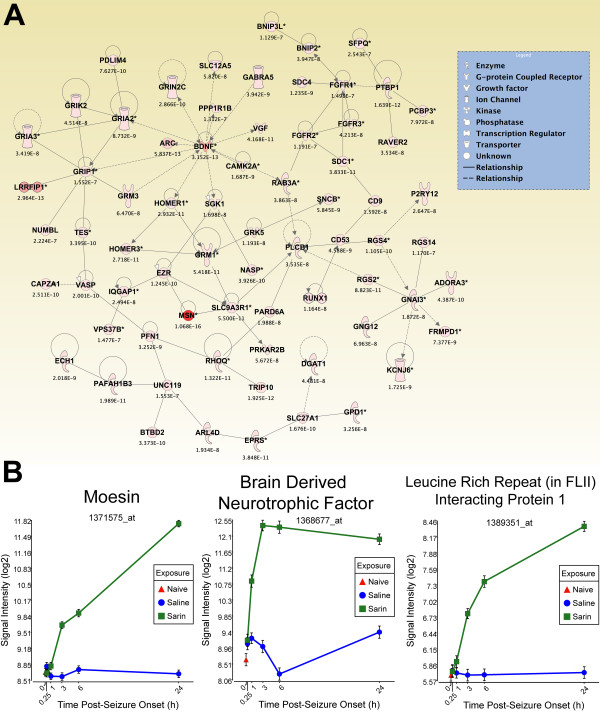
**Seizure-induced alteration of cell-to-cell signalling/interaction, nervous system development/function, and cell morphology gene network**. As detailed in Figure 5, a two-way ANOVA was performed to identify genes most significantly changed based on exposure and time after seizure onset. Genes meeting a p-value cutoff of 3.046 × 10^-7 ^were overlaid onto a global molecular network developed from information within the IPA Knowledge Base, and the networks were then algorithmically generated based on their connectivity. (A) Graphical representation of the network reveals significant changes in gene expression due to sarin-induced seizure. Genes are represented as nodes of various shapes to represent the functional class of the gene product, and the biological relationship between two nodes is represented as a line. The intensity of the node color indicates the degree of differential expression. (B) Top three genes within network significantly altered by sarin-induced seizure. Moesin (MSN) and leucine rich repeat (in FLII) interacting protein 1 (LRRFIP1) expression increased over the time course with peak expression at 24 h after seizure onset, while brain derived neurotophic factor (BDNF) expression peaked at 3 h after seizure onset and dropped slightly at 6 and 24 h.

To generate an overall view of the significant molecular effects of sarin-induced seizure over the 24-h time course, a two-way interaction ANOVA was performed using exposure (saline vs. sarin) and time as factors. Gene ontology analysis identified biological functions associated with cellular movement, cellular growth and proliferation, and inflammatory response as being the most significant (see Additional File 18). Additionally, the most significant canonical pathways were IL-6 signaling, IL-10 signaling, TREM1 signaling, MIF regulation of innate immunity, type I diabetes mellitus signaling, p38 MAPK signaling, toll-like receptor signaling, and acute phase response signaling (see Additional File 19). In addition to the canonical pathways contained in the IPA Knowledge Base, we also assessed the five *de novo *networks of genes most significantly modulated by sarin-induced seizure. In agreement with the findings reported above, one of the most significant networks identified was built around transforming growth factor-β (TGF-β) as a central node and was associated with cell-to-cell signaling and interaction, inflammatory response, and cellular movement (Figures 5 and 6). Another significant network was built around jun oncogene (JUN) as a central node and was associated with cell death, cellular development, and cellular function and maintenance (Figures 7 and 8). The third network was constructed around MAPK1 and was associated with cellular movement, cell death, and cell morphology (Figures 9 and 10). The fourth network, built around cyclin-dependent kinase inhibitor 1A (CDKN1A), was associated with cellular growth and proliferation, cellular development, and lipid metabolism (Figure 11). The fifth network was primarily focused on brain-derived neurotrophic factor (BDNF) and was associated with cell-to-cell signaling and interaction, nervous system development and function, and cell morphology (Figure 12).

### Multiplexed RT-PCR validation of microarray analysis data

The GeXP genetic analysis system was used to validate a subset of differentially expressed genes. The capillary electrophoresis-based system was used to separate multiplexed RT-PCR reactions to compare the expression levels of 21 inflammatory cytokines and chemokines in each RNA sample. The subset of genes showed relative differences in expression level following sarin-induced seizure that corresponded to expression changes seen in the microarray analysis (see Additional File 20).

Using both technologies, IL-1α, CCL4, TNF-α, and CCL3 expression appeared to be up-regulated in all five time points, peaking at 3 hr post-seizure onset. CCL7, IL-6, and IL-1ß were also up-regulated at all five time points using both methods. CCL7 peaked at 24 h, IL-1ß peaked at 1h and 24 h, and IL-6 peaked at 3 and 24 h. Nampt and CCL2 were up-regulated at all time points using both approaches, with expression peaking at 6 h using multiplexed PCR analysis and 24 h using microarray analysis. Conversely, SCG2 expression appeared to peak at 6 h using microarray analysis, but appeared to peak at 3 h with multiplexed PCR analysis. In both analyses, CXCL16 and IL-18 were down-regulated at 0.25 h post-seizure onset and up-regulated throughout the remainder of the 24 h time course. SPP1 also displayed the same expression pattern with the exception that it appeared to be down-regulated at 1 h using the multiplexed PCR method. CCL17 expression level also increased over the 24-h time course using microarray analysis, but there appeared to be a slight decrease in expression at 1 h in the multiplexed PCR analysis. SPRED2 showed the same relative pattern of expression changes over the time course but appeared to be slightly more down-regulated using the multiplexed PCR method. CNTF also appeared to be more down-regulated using the multiplexed PCR method and had a slightly different pattern in expression changes over the time course. CXCL10 was down-regulated at 0.25 and 1 h with both assays and appeared to peak in expression at 6 h. CXCL12 and CTF1 were down-regulated at all time points using both assays. MIF expression was also down-regulated at all time points in microarray analysis, but was slightly up-regulated at 1 h in the multiplexed PCR analysis. CXCL1 displayed a different pattern of expression between the two methods. Using microarray analysis, it appeared to be up-regulated in all five time points and peaked at 3 and 24 h. With the multiplexed PCR methodology, it showed to be slightly down-regulated at 0.25 h and peaked at 6 h.

Therefore, the overall expression levels of the examined inflammatory cytokines and chemokines were in close agreement using the two different methodologies. The differences in expression levels can easily arise from variation in quantitative methodology. Because these two technologies are different, slight differences in expression between the two methods are expected, but overall the data between the microarray analysis and multiplexed PCR analysis are in close agreement.

## Discussion

Despite extensive efforts to prevent their use, OP nerve agents remain a viable threat to soldiers in times of war as well as to the civilian population in the event of a terrorist attack. It is well-established that the acute toxicity of nerve agents is the result of AChE inhibition; however, the molecular mechanisms and biological pathways involved in the resulting neurodegeneration following seizure onset are poorly understood. To help determine these molecular events, animals were challenged with a 1 × LD_50 _dose of sarin, and microarray analysis was used to identify gene expression changes in the rat piriform cortex over a 24-h time period following seizure onset. Using our exposure model, roughly 50% of the sarin-exposed animals developed seizure activity with a mean latency of 10.2 min, as indicated by behavioral assessment and electrocortical activity. In this study, we analyzed gene expression changes in the piriform cortex of seizing animals because it has been identified as one of the regions in the central nervous system to show massive, early-onset tissue pathology following nerve agent-induced seizures [10,13,25]. In agreement with previous studies [6,15,26,28,36], we found major gene expression profile differences correlated with seizure induction and identified a strong inflammatory response that could potentially lead to brain injury and cell death. The transcriptional responses of sarin-exposed non-seizing animals in this dataset will be the focus of a future manuscript.

Many significant molecular changes were seen at our earliest time point of 0.25 h after seizure onset. Unlike the later time points, a number of these changes were seen in metabolic pathways, such as those involved in the metabolism of glutamate, inositol, and phospholipids. The number of significantly altered genes and canonical pathways increased over time following seizure induction and appeared to represent an inflammatory response, which was seen as early as 0.25 h. This is suggested by the appearance of biological functions such as cell-mediated immune response (0.25 h, 1 h, 3 h, 6 h), immune cell trafficking (0.25 h, 1 h, 6 h, 24 h), inflammatory response (0.25 h, 1 h, 6 h, 24 h), and immunological disease (1 h, 3 h, 24 h) among the 25 biological functions most significantly altered following sarin-induced seizure. Further support of this statement is provided by the number of inflammatory-related pathways that were significantly altered at each time point, such as IL-10 signaling (0.25-24 h), PPAR signaling (0.25-24 h), IL-6 signaling (1-24 h), and TREM1 signaling (1-24 h). Thus, even at our latest time point of 24 h, inflammatory functions and pathways were still significantly altered by sarin-induced seizure.

In addition to our analysis of molecular effects at each time point, we also performed a two-way interaction ANOVA using exposure (saline vs. sarin) and time to obtain an overall view of significant molecular effects resulting from sarin-induced seizure during the 24-h time course. In agreement with the analyses performed at each individual time point, we identified biological functions associated with an inflammatory response (e.g., inflammatory response, inflammatory disease, immune cell trafficking, and immunological disease) among the most significant responses. Additionally, the most significant canonical pathways identified were all related to an inflammatory response. These included IL-6 signaling, IL-10 signaling, TREM1 signaling, MIF regulation of innate immunity, type I diabetes mellitus signaling, p38 MAPK signaling, toll-like receptor signaling, and acute phase response signaling. In our assessment of the five *de novo *networks of genes most significantly modulated by sarin-induced seizure over the 24-h time course, we identified those associated with cell-to-cell signaling and interaction, inflammatory response, cellular movement, cell death, cellular development, cellular function and maintenance, cell morphology, cellular growth and proliferation, lipid metabolism, and nervous system development and function.

The findings presented in this study support the temporal model proposed by McDonough and Shih [6] that links nerve agent-induced seizures to resulting neuropathology. In this three-phase model, seizure initiation is a cholinergic phenomenon that lasts from the time of exposure to approximately 5 min after seizure onset. It has been reported that a convulsant dose of soman immediately inhibits brain cholinesterase with maximum inhibition within 10 min and a large increase in ACh concentration at the time of seizure initiation. Furthermore, previous studies have shown an immediate induction of AChE mRNA expression levels in the rat brain following sarin exposure [37,38]. If seizures are not immediately stopped, a transition phase occurs 5-40 min post-exposure where other neurotransmitter systems are perturbed. During this phase, the level of excitatory amino acids (EAAs), such as glutamate, increases and potentiates seizure activity [6]. Our findings at 0.25 h after seizure onset support this transition phase of the model. D-glutamine and D-glutamate metabolism and glutamate receptor signaling were two of the canonical pathways significantly altered immediately after seizure onset. Previous studies have shown an increase in choline (Ch), a precursor for ACh, 15-30 min after nerve agent exposure [39], a time in which seizure activity has already been initiated, due to increased hydrolysis of phospholipids [40], and is supported by the presence of phospholipid degradation among the significant pathways at this early time point. The most significantly altered pathway at 0.25 h was inositol metabolism. Inositol works closely with Ch as a primary component of cell membranes. It is necessary for normal nerve and brain function as it is required for proper action of several neurotransmitters, such as ACh and serotonin. Studies have shown that membrane phosphoinositide (PI) is hydrolyzed following the activation of neurotransmitter receptors, such as N-methyl-D-aspartate (NMDA), to yield inositol 1,4,5-triphosphate (IP_3_), a second messenger that transmits signals from the receptor into the cell by releasing calcium from non-mitochondrial intracellular stores [6,8,41]. This leads to the last phase of the model, which is predominantly a noncholinergic phenomenon starting approximately 40 min after seizure onset with the presence of prolonged epileptiform activity. It is proposed that this excess influx of calcium is the ultimate cause of neuropathology following nerve agent exposure as it can hyperactivate enzymes such as lipases, proteases, endonucleases, kinases, or phosphatases that can cause damage to cell membranes, cytoskeleton, or organelle structure and function [41,42].

Since the development of the model proposed by Shih and McDonough, many studies have shown that there is also an increase in pro-inflammatory mRNA and protein expression following nerve agent exposure that lasts hours-to-days after exposure [24,27,29-31]. Furthermore, there has been increasing evidence over the past several years implicating inflammatory reactions in the pathogenesis of several neurodegenerative disorders, such as Alzheimer's disease, Parkinson's disease, multiple sclerosis, and epilepsy [43,44]. Studies using various seizure models have shown an increase in cytokine mRNA and protein expression levels within 30 min following seizure induction in brain regions involved in seizure onset and spread [45-47]. Therefore, it is likely that this late phase of the model involves neuroinflammatory processes that lead to neuropathology following nerve agent exposure.

Pro-inflammatory cytokines, such as IL-1β, IL-6, and TNF-α, are expressed at very low levels in healthy brain tissue but are rapidly induced following insult. In our study, we observed a significant increase in pro-inflammatory gene expression as early as 0.25 h following sarin-induced seizure onset, and this inflammatory response was still present at our latest observed time point of 24 h. In support of this finding, Damodaran et al. [22,23] and Chapman et al. [29] also observed an increase in cytokine expression following sarin-induced seizure activity. Damodaran et al. [22,23] used microarray analysis to study gene expression profiles 0.25 h after sarin exposure. As with our study, they reported numerous changes in gene expression profiles immediately following sarin exposure with cytokines being among the significantly altered signal transduction pathways. Chapman and colleagues [29] used midazolam to control seizure duration and monitored protein expression levels of IL-1β, IL-6, TNF-α, and prostaglandin E2 (PGE2) in the hippocampus and cortex at 2, 4, 6, 8, 24, 48, and 144 h and 30 days following 5 or 30 min of seizure activity. They observed a significant increase in cytokine expression starting at their earliest time point of 2 h and peaking at 2-24 hr following sarin, with the greatest increase in animals subject to 30 min of seizure activity.

Further support of our findings is provided by studies that show a neuroinflammatory response to soman. Svensson et al. [26,28] have previously shown increases in IL-1β mRNA and protein levels following soman exposure. In addition, Dhote et al. [30] and Williams et al. [27] used quantitative RT-PCR to analyze the neuroinflammatory gene response following a convulsant dose of soman (1.6 × LD_50_). Dhote et al. [30] showed an increase of IL-1B, TNF-α, IL-6, inter-cellular adhesion molecule-1 (ICAM-1), and suppressor of cytokine signaling (SOCS) 3 mRNA in the whole cortex at 0.50, 1, 2, 6, 24, 48, and 168 h following soman exposure, which confirmed the earlier findings of Williams et al. [27] where they observed an initial up-regulation of TNF-α mRNA at 2 h post-exposure followed by an increase in IL-1β and IL-6 mRNAs 6 h later. Johnson and Kan [31] have recently quantified the protein levels of these cytokines in vulnerable brain regions following soman-induced seizure onset. They reported a significant increase in IL-1β, IL-6, and TNF-α protein levels between 10-18 hrs after the mRNA peak expression levels. Dillman et al. [24] used oligonucleotide arrays to analyze gene expression profiles of rat hippocampi at 1, 3, 6, 12, 24, 48, 72, 96, and 168 h following exposure to a convulsant dose of soman. In agreement with our findings, they observed an increasing alteration in gene expression profiles over the first 24 h following soman exposure. Within this time frame, they identified a strong inflammatory response with the presence of immunological and inflammatory disease among the most significant biological processes altered, and the most significant canonical signaling pathways including p38 MAPK, toll-like receptor, IL-6, and IL-10. During the later phase of their time course, they observed a shift in expression that resembled an injury response (24-96 h), which was subsequently followed by a recovery phase at their latest time point of 168 h. The similarities during the first 24 of our study and the study done by Dillman and colleagues [24] lead us to believe that we would also observe a similar shift in gene expression that would involve molecular processes and pathways involved in an injury and recovery phase. However, further studies analyzing gene expression profiles over a longer time period are needed to confirm this same mechanism of action following sarin-induced seizure. Angoa-Pérez and colleagues [11] recently studied the effects of soman on the expression of cyclooxygenase-2 (COX-2), which is the initial enzyme in the biosynthetic pathway of pro-inflammatory prostaglandins (PGEs) and a factor that has been implicated in seizure initiation and propagation. They found that the induction of COX-2 expression and subsequent production of PGEs correlated with seizure intensity in the rat brain from 4 h to 7 d, suggesting that these molecules could play a role in neuronal degeneration well after the cholinergic and glutamatergic response. Angoa-Pérez and colleagues hypothesize that seizures occurring in response to a PGE overload would likely not respond to the standard treatment of anticholinergics and benzodiazepines, indicating that other therapeutics, such as COX-2 inhibitors, should be added to prevent or minimize neuropathology that occurs in the later phase of the McDonough and Shih model.

## Conclusions

This analysis of gene expression profiles following sarin-induced seizure supports previous findings of mRNA and protein alterations following OP nerve agent intoxication. It also provides further evidence for the presence of non-cholinergic and non-glutamatergic systems during the late phase response to nerve agents that was proposed by McDonough and Shih in 1997 [6]. In addition, previous studies using AChE knockout mice have further proved the presence of non-AChE targets for OP nerve agents [48]. The rapid and persistent alteration of pro-inflammatory cytokines seen in this study as well as previous studies strongly suggests that they may play a causal role in long-term pathological changes following exposure to OP nerve agents. Therefore, antagonism of pro-inflammatory molecules, as well as their receptors and signaling pathways, may represent a new approach for the development of additional therapies to better protect the brain against seizure-induced damage. Several seizure-inducing sites have been identified within the piriform cortex [10,13,25], so it seems logical to focus on the molecular alterations in this brain region to help identify these potential molecular targets. Because current countermeasures may not fully prevent neurological damage, this type of in-depth analysis is critical to examine the molecular effects following nerve agent exposure and identify therapeutics that can reduce or block the cascade of secondary events that lead to neuropathology and associated functional impairments. Furthermore, the identification of efficacious drug treatments that reduce pathology in our nerve agent model may have implications for potential therapies in epilepsy and other neurodegenerative disorders such as Alzheimer's disease, Huntington's disease, and Parkinson's disease.

## List of abbreviations

2-PAM: 2-pyridine aldoxime methylchloride; OP: organophosphorus; CWA: chemical warfare agent; AChE: acetylcholinesterase; ACh: acetylcholine; IED: improvised explosive device; EEG: electroencephalogram; RMA: robust multiarray averaging; ANOVA: analysis of variance; FDR: false discovery rate; IPA: Ingenuity Pathways Analysis; PCA: principal component analysis; IL: interleukin; TRK: tyrosine kinase receptor; PPAR: peroxisome proliferator-activated receptor; TREM1: triggering receptor expressed on myeloid cells 1; TNF-α: tumor necrosis factor-α; MAPK: mitogen activated protein kinase; HMGB1: high-mobility group protein 1; GM-CSF: granulocyte-macrophage colony-stimulating factor; NF-κB: nuclear factor kappa-light-chain-enhancer of activated B cells; GNRH: gonadotropin-releasing hormone; ATM: ataxia telangiectasia mutated protein; NRF2: nuclear factor erythroid 2-related factor 2; ILK: integrin-linked kinase; MIF: macrophage migration inhibitory factor; TGF-β: transforming growth factor-β; JUN: jun oncogene; CDKN1A: cyclin-dependent kinase inhibitor 1A; EEA: excitatory amino acid; Ch: choline; PI: phosphoinositide; NMDA: N-methyl-D-aspartate; IP_3_: inositol 1,4,5-triphosphate; PGE2: prostaglandin E2; ICAM-1: inter-cellular adhesion molecule-1; SOCS: suppressor of cytokine signalling; COX-2: cyclooxygenase-2; PGE: prostaglandin; CCL3: chemokine (C-C motif) ligand; TNFAIP6: tumor necrosis factor alpha induced protein 6; SPRED2: sprouty-related, EVH1 domain containing 2; CNTF: ciliary neuronotrophic factor; SCG2: secretogranin-2; CXCL: chemokine (C-X-C) motif ligand; SPP1: secreted phosphoprotein 1; Nampt: nicotinamide phosphoribosyltransferase; CTF1: cardiotrophin-1

## Competing interests

The authors declare that they have no competing interests.

## Authors' contributions

KDS collected and processed brain regions for microarray and multiplexed PCR analysis, analyzed all data, and drafted the manuscript. LAL participated in developing and coordinating the study. CLR conducted behavioral assessments of the animals. JLM dissected brain regions from all animals. JFD participated in the brain dissections, study design, data analysis, and helped draft the manuscript. All authors read and approved the final manuscript.

## Supplementary Material

Additional file 1 **Gene symbol, PCR product size, and primer sequences used in multiplex RT-PCR assays**. Click here for file

Additional file 2 **Concentrations of reverse primers within the RT-PCR multiplex**. Click here for file

Additional file 3 **Top ≤ 800 genes altered at 0.25 h post-seizure onset *(p-*value cutoff ≤ 0.05)**. Click here for file

Additional file 4 **Top ≤ 800 genes altered at 1 h post-seizure onset *(p-*value cutoff ≤ 4.35 × 10^-2^)**. Click here for file

Additional file 5 **Top ≤ 800 genes altered at 3 h post-seizure onset *(p-*value cutoff ≤ 4.66 × 10^-3^)**. Click here for file

Additional file 6 **Top ≤ 800 genes altered at 6 h post-seizure onset *(p-*value cutoff ≤ 2.54 × 10^-3^)**. Click here for file

Additional file 7 **Top ≤ 800 genes altered at 24 h post-seizure onset *(p-*value cutoff ≤ 6.25 × 10^-4^)**. Click here for file

Additional file 8 **Top 25 biological functions identified 0.25 h after seizure onset in piriform cortex**. The top ≤ 800 molecules from the dataset that met the p-value cutoff (≤ 0.05) and were associated with biological functions and/or diseases in the IPA Knowledge Base were considered for the analysis. Right-tailed Fisher's exact test was used to calculate the *p*-value determining the probability that each biological function and/or disease assigned to the dataset was due to chance alone. The categories listed in the table refer to a high level function within the IPA Knowledge Base. Each high level function can be further broken down into multiple specific functions (e.g., the category inflammatory response is comprised of individual functions such as chemotaxis, recruitment, acute phase reaction, and phagocytosis). Therefore, the range of significance values refers to the individual functions contained within the category listed. The calculated *p*-values for each category are expressed as -log (*p*-value), so values greater than 1.3 indicate significance. Click here for file

Additional file 9 **Top 25 biological functions identified 1 h after seizure onset in piriform cortex**. Click here for file

Additional file 10 **Top 25 biological functions identified 3 h after seizure onset in piriform cortex**. Click here for file

Additional file 11 **Top 25 biological functions identified 6 h after seizure onset in piriform cortex**. Click here for file

Additional file 12 **Top 25 biological functions identified 24 h after seizure onset in piriform cortex**. Click here for file

Additional file 13 **Significant canonical pathways identified 0.25 h after seizure onset in piriform cortex**. The top ≤ 800 molecules from the dataset that met the p-value cutoff (≤ 0.05) and were associated with a canonical pathway in IPA's Knowledge Base were considered for the analysis. The significance value for each canonical pathway was calculated using right-tailed Fisher's exact test and is expressed as -log (*p*-value), so values greater than 1.3 indicate significance. Click here for file

Additional file 14 **Significant canonical pathways identified 1 h after seizure onset in piriform cortex**. Click here for file

Additional file 15 **Significant canonical pathways identified 3 h after seizure onset in piriform cortex**. Click here for file

Additional file 16 **Significant canonical pathways identified 6 h after seizure onset in piriform cortex**. Click here for file

Additional file 17 **Significant canonical pathways identified 24 h after seizure onset in piriform cortex**. Click here for file

Additional file 18 **Top 25 biological functions affected in piriform cortex from 0.25 h to 24 h after seizure onset**. Click here for file

Additional file 19 **Significant canonical pathways affected in piriform cortex from 0.25 h to 24 h after seizure onset**. Click here for file

Additional file 20 **DNA microarray analysis and Multiplexed RT-PCR show similar changes in gene expression following sarin-induced seizure**. The GeXP genetic analysis system was used to measure the expression levels of 21 differentially expressed cytokines or chemokines by multiplexed RT-PCR to validate the microarray data. The expression of each gene within a sample was normalized to GAPDH expression to minimize inter-capillary variation, and the normalized intensity of each replicate (n ≥ 3) was used to calculate an average intensity of each sample group (i.e. control or sarin-induced seizure at each time point). The fold expression difference between control and sarin-induced seizure samples is shown for each gene at each of the five time points examined. The fold changes in expression obtained in the microarray analysis are shown on the left, and the fold changes in expression obtained in the multiplexed PCR analysis are shown on the right. Genes that were down-regulated following sarin-induced seizure are shaded in green, and genes that were up-regulated following sarin-induced seizure are shaded in red. Click here for file
