# Supplementary figures and images for: Transcriptional analysis of rat piriform cortex following exposure to the organophosphonate anticholinesterase sarin and induction of seizures
Source: J Neuroinflammation. 2011 Jul 21;8:83. doi: 10.1186/1742-2094-8-83 (PMC3199787; doi:10.1186/1742-2094-8-83)

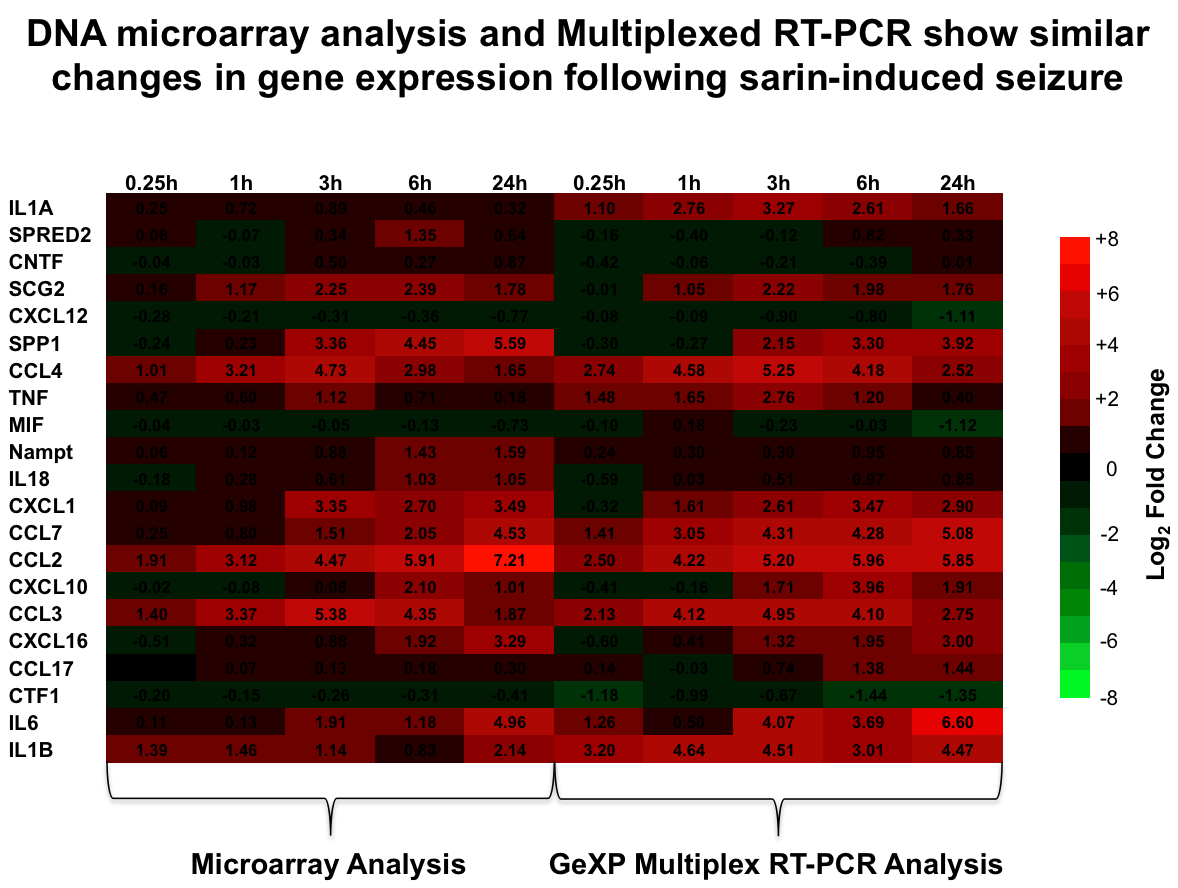

Supplement: Additional file 20 — DNA microarray analysis and Multiplexed RT-PCR show similar changes in gene expression following sarin-induced seizure. The GeXP genetic analysis system was used to measure the expression levels of 21 differentially expressed cytokines or chemokines by multiplexed RT-PCR to validate the microarray data. The expression of each gene within a sample was normalized to GAPDH expression to minimize inter-capillary variation, and the normalized intensity of each replicate (n ≥ 3) was used to calculate an average intensity of each sample group (i.e. control or sarin-induced seizure at each time point). The fold expression difference between control and sarin-induced seizure samples is shown for each gene at each of the five time points examined. The fold changes in expression obtained in the microarray analysis are shown on the left, and the fold changes in expression obtained in the multiplexed PCR analysis are shown on the right. Genes that were down-regulated following sarin-induced seizure are shaded in green, and genes that were up-regulated following sarin-induced seizure are shaded in red. [file 1742-2094-8-83-S20.PNG]
